# Multimodal Federated Learning: A Survey

**DOI:** 10.3390/s23156986

**Published:** 2023-08-06

**Authors:** Liwei Che, Jiaqi Wang, Yao Zhou, Fenglong Ma

**Affiliations:** 1 College of Information Sciences and Technology, Pennsylvania State University, University Park, PA 16802, USA; lfc5481@psu.edu (L.C.); jqwang@psu.edu (J.W.); 2 Instacart, San Francisco, CA 94105, USA

**Keywords:** federated learning, multimodal learning, Internet of Things

## Abstract

Federated learning (FL), which provides a collaborative training scheme for distributed data sources with privacy concerns, has become a burgeoning and attractive research area. Most existing FL studies focus on taking unimodal data, such as image and text, as the model input and resolving the heterogeneity challenge, i.e., the challenge of non-identical distribution (non-IID) caused by a data distribution imbalance related to data labels and data amount. In real-world applications, data are usually described by multiple modalities. However, to the best of our knowledge, only a handful of studies have been conducted to improve system performance utilizing multimodal data. In this survey paper, we identify the significance of this emerging research topic of multimodal federated learning (MFL) and present a literature review on the state-of-art MFL methods. Furthermore, we categorize multimodal federated learning into congruent and incongruent multimodal federated learning based on whether all clients possess the same modal combinations. We investigate the feasible application tasks and related benchmarks for MFL. Lastly, we summarize the promising directions and fundamental challenges in this field for future research.

## 1. Introduction

In various real-world scenarios, data are usually collected and stored in a distributed and privacy-sensitive manner—for instance, multimedia data on personal smartphones, sensory data from various vehicles, and examination data and diagnostic records of patients across different hospitals. The significant volume of sensitive yet multimodal data being collected and shared has heightened people’s concerns regarding privacy protection. Consequently, there has been an emergence of increasingly stringent data regulation policies, such as the General Data Protection Regulation (GDPR) in the European Union and the Health Insurance Portability and Accountability Act (HIPAA) in the United States. These regulations have given rise to challenges in data collaboration and have raised privacy concerns for traditional centralized multimodal machine learning approaches [1].

**Figure 1:**
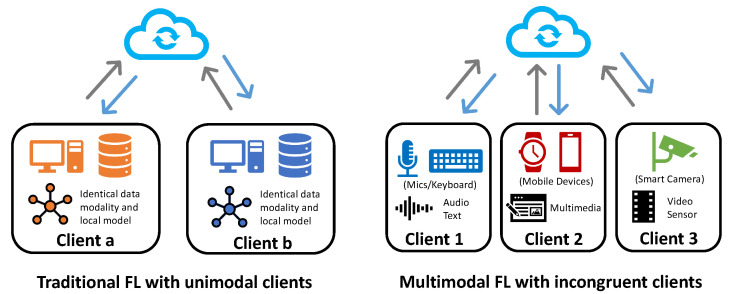
Illustration of traditional unimodal FL v.s. multimodal FL.

To address these data privacy concerns, a novel paradigm called federated learning (FL) [2] has been introduced. This approach enables distributed clients to collaboratively train a high-performing global model without sharing their local data, effectively preventing privacy leakage through data transmission. However, the majority of previous works have focused on the unimodal setting, where all the clients in the federated system hold the same data modality, as shown in Figure 1 (left). Among these studies, statistical heterogeneity [3], i.e., the non-IID challenge, caused by the skew of labels, features, and data quantity among clients, is one of the most critical challenges that has attracted much attention [4,5,6,7,8]. In contrast, multimodal federated learning, as shown in Figure 1 (right), further introduced the modality heterogeneity challenge, which led to significant differences in model structures, local tasks, and parameter spaces among clients, thereby exposing the substantial limitations of traditional unimodal algorithms.

Federated systems trained with multimodal data are intuitively more powerful and insightful compared to unimodal ones [1]. We define the modality types held by the clients as their modality combinations, which determine the local tasks they perform. If two clients hold the same or similar modality combinations (e.g., both image and text data), they have a smaller semantic gap and task gap. In other words, the more congruent modality combinations the clients hold, the less heterogeneous the modality distribution of the system.

**Figure 2:**
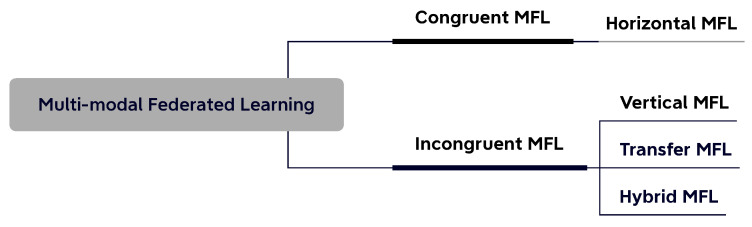
Taxonomy of multimodal federated learning (MFL).

Based on the congruence of modality distribution, MFL can be divided into two categories: congruent MFL and incongruent MFL, as depicted in Figure 2. In ***congruent MFL***, the clients hold similar or the same local modality combinations, and horizontal FL is the typical setting of this type. The majority of existing MFL work [9,10,11,12] has also focused on this federated setting, where all the clients hold the same input modality categories and feature space but differ as to the sample space. In [10], the authors proposed a multimodal federated learning framework for multimodal activity recognition with an early fusion approach via local co-attention. The authors in [12] provided a detailed analysis of the convergence problem of MFL with late fusion methods under the non-IID setting. In the healthcare domain [13,14,15], congruent MFL has shown great application value by providing diagnosis assistance with distributed digital health data.

For ***incongruent MFL***, the clients usually hold unique or partially overlapped data modality combinations, which makes the federated optimization and model aggregation more challenging. This category contains vertical multimodal federated learning (VMFL), multimodal federated transfer learning (MFTL), and hybrid multimodal federated learning (hybrid MFL). In VMFL, the clients hold different input modalities and feature spaces, but all the data samples are in the same space. In [16], the authors assumed that each client only held one specific modality and, correspondingly, proposed FDARN, a five-module framework, for cross-modal federated human activity recognition (CMF-HAR). For MFTL, the clients mainly differ as to feature spaces (e.g., photographic images and cartoon images) and sample ID spaces. For instance, in [17], the authors proposed a fine-grained representation block named aimNet. They evaluated their methods under different FL settings, including the transfer setting between two different vision–language tasks.

Hybrid MFL is a more challenging setting, where the data relationships among the clients cannot be appropriately described by any of the above three settings alone. The clients in a hybrid setting can hold different local data, varying in terms of both modality categories and quantities. Given *M* modalities in a federated system, the theoretical client types are $2^{M}-1$, including both unimodal and multimodal clients. Ref. [18] discussed a significant challenge for hybrid MFL, i.e., modality incongruity, where the unique modality combination among the clients enlarges the heterogeneity. They proposed FedMSplit for multitask learning in the hybrid MFL setting, with a graph-based attention mechanism to extract the client relationship for aggregation.

Based on our observation of the increasing interest among researchers in exploring the challenges of multimodal data in FL [10,11,15,16,19,20], multimodal federated learning has emerged as a promising and practical topic with numerous application scenarios. However, much of the research in this area has been conducted in customized multimodal federated learning scenarios, lacking categorization and standardization. The diverse and varied nature of this field emphasizes the need for a systematic investigation and study on multimodal federated learning topics. Therefore, we present our perspective on exploring multimodal data in federated learning and outline our contributions below:

- We conducted a comprehensive literature review on existing multimodal federated learning research, leading to the formal definition of multimodal federated learning (MFL). We also introduced essential concepts like modality combination and modality heterogeneity, which distinguish MFL from traditional FL.
- To enhance the clarity and organization of the field, we classified existing MFL work into four categories: horizontal, vertical, transfer, and hybrid MFL. By expanding upon traditional unimodal federated learning, this categorization provides a structured framework for the design and development of subsequent MFL research, facilitating method comparison and advancement.
- Given the current lack of well-defined evaluation benchmarks for MFL, we thoroughly examined feasible application scenarios of MFL and surveyed relevant and suitable open-source benchmark datasets that can serve as valuable resources for researchers in this domain.
- We identified and summarized significant challenges and potential research directions in MFL, shedding light on unique issues such as modality heterogeneity and missing modalities. These insights offer valuable guidance for future research and innovation in the field of multimodal federated learning.

The rest of this paper is organized as follows. We introduce the methodology used to conduct the literature review in Section 2. In Section 3, we summarize the three popular aspects for mitigating the statistical heterogeneity in unimodal federated learning systems. In Section 4, we present preliminaries and a formal definition of multimodal federated learning. In Section 5, we categorize multimodal federated learning into four types based on the input modalities of the clients. We introduce the common tasks and benchmarks for MFL in Section 6 and Section 7, respectively. Section 8 identifies the challenges and promising research directions, as well as the potential application scenarios.

## 2. Methodology

The exploration of multimodal data in federated learning is still in its nascent stage. Below, we introduce the process we followed to collect and analyze the related papers.

### 2.1. Search Strategy

We identified related literature in the multimodal federated learning field by performing comprehensive searches in multiple databases including IEEE Xplore, ACM Digital Library, Google Scholar, and arXiv. Specifically, we used the following keywords in combination to ensure a broad and inclusive search. The keywords of the search queries included “multimodal federated learning”, “cross-modal federated learning”, “collaborative learning”, “multimodal data”, and their combinations. The search was conducted up to 23 June 2023, and all papers satisfying our search criteria until that date were considered for inclusion in this survey.

### 2.2. Search Criteria

To ensure the relevance and quality of the papers included in this survey, specific inclusion and exclusion criteria were applied during the screening process. We included the paper if it satisfied all the following criteria:

1. Pertinent to multimodal federated learning or closely related topics;
2. Written in English;
3. Published in peer-reviewed conferences or journals;
4. Full-text access;
5. Clear cross-validation and comparison with related literature;
6. Evaluation on the rigor of the research methodologies and the significance of the contributions.

### 2.3. Screening Process

**Figure 3:**
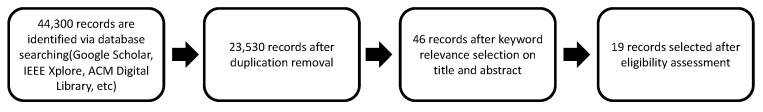
A diagram of the screening process.

As shown in Figure 3, the literature search and selection process was conducted via two stages. In the first stage, we obtained 44,300 records according to the keyword-based search in different databases. We removed 20,770 records due to duplication. According to the relevance of the titles and abstracts, we further selected 46 papers for the second stage of the screening by reviewing the paper content. Finally, we selected 19 papers for our review purpose based on the inclusion criteria.

## 3. Federated Learning for Unimodal Heterogeneity

To mitigate the optimization divergence caused by heterogeneity challenges and increase the system robustness under the non-IID setting, existing unimodal methods have mainly been proposed from three perspectives: federated convergence optimization, personalized federated learning, and federated multitask learning.

### 3.1. Federated Convergence Optimization

From the convergence optimization perspective, Zhao et al. in [4] investigated the under-performance problem of FedAvg [2] under the non-IID setting. They demonstrated that weight divergence caused the performance reduction issue and provided further analysis on this subject. In [21], the authors discovered that non-IID data caused the problem of client drift in the uploading process and affected the system convergence rates. FedProx [22] modifies the local objective of each client, adding a proximal term to it. In [23], the authors proposed SCAFFOLD to mitigate the client drifts between the local models and the global model. In [24], FedBVA decomposed the aggregation error into bias and variance for collaborative adversarial training in order to improve convergence and performance. In [6], the authors introduced reinforcement learning into the federated global update stage, where the server dynamically selected a subset of clients with the highest rewards to mitigate the heterogeneity challenge. The mutual target of these studies was to mitigate the divergence or drift problem during global updating so that the framework could achieve a more generalized global model.

### 3.2. Personalized Federated Learning

Personalized federated learning [25] is a research topic proposed to handle the statistical heterogeneity challenge (i.e., the non-IID setting) from another perspective, and many existing methods aim to provide each client with personalized adaptation instead of a one-fits-all global solution. Ruan and Joe-Wong in [26] proposed FedSoft, which reduced the clients’ workload using proximal updates and brought both personalized local models and global cluster models. PerFedAvg [27] adapted meta-learning into the FL framework, where it treated the global model as a meta-model to provide a few-shot adaptation for each client. In [28], the authors added Moreau envelopes as a regularization term in the local loss functions to help achieve personalized model optimization. In [29], the authors reassembled models and selected the most fitted personalized models for clients by calculating the similarity.

### 3.3. Federated Multitask Learning

Instead of seeking a general solution, federated multitask learning [30] aims to find personalized models for each client by utilizing the similarity among them. MOCHA [30] was the first proposed federated multitask learning framework for convex models. Corinzia et al. in [31] proposed VIRTUAL, which used approximated variational inference and simulated the federated system with a star-shaped Bayesian network. Marfoq et al. in [32] used a federated expectation-maximization algorithm to solve the multitask problem with a mixture of unknown underlying distributions.

The existing methods have shown great achievements in alleviating the statistical heterogeneity challenge via global optimization or learning personalized local models. However, as the heterogeneity among different client distributions diverges, the unified global model may not exist. Especially in the multimodal setting, federated clients could have different local model structures due to the modality heterogeneity, which makes the general solution more unfeasible. Local model personalization could also be inapplicable, due to the differences in both feature space and parameter space for MFL. In such a case, exploring multimodal data under the federated learning paradigm cannot rely on a direct combination of the existing techniques. Instead, the introduction of modality heterogeneity among the clients brings unique obstacles and makes the existing challenge even more demanding.

## 4. Preliminaries of Multimodal Federated Learning

Compared to unimodal federated learning, we define multimodal federated learning as federated systems containing at least two data modalities among all the local datasets. In the following, we will formally define multimodal federated learning using the multimodal classification task as an example.

In a multimodal federated learning system, given *K* clients and a modality category number *M*, where K≥2 and M≥2, each client *k* is assumed to have access to a local dataset $D_{k}$, which contains a group of data sample IDs. The size of the dataset is determined by the number of sample IDs, i.e., $|D_{k}|$; $M_{k}$ represents the total data modality number for client *k*; and $M_{k}\in \left[1,M\right]$. If $M_{k}=1$, client *k* is called the *unimodal client*, and it is a *multimodal client* if $M_{k}>1$.

To identify the types of clients based on their local data modalities, we define the local multimodal dataset $D_{k}$ of an arbitrary client *k* as

(1) $$D_{k}={\left\{{\left(x_{k}^{m_{1}},x_{k}^{m_{2}},\ldots ,x_{k}^{m_{M_{k}}},y_{k}\right)}_{i}\right\}}_{i=1}^{|D_{k}|},$$

where $x_{k}^{m}$ represents a data sample of *m*-modality in client *k*. The *i*-th data sample of the *k*-th local dataset is $X_{k}\left(i\right)={\left(x_{k}^{m_{1}},x_{k}^{m_{2}},\ldots ,x_{k}^{m_{M_{k}}}\right)}_{i}$. The modality combination of this local set is defined as $X_{k}=\left(m_{1},m_{2},\ldots ,m_{M_{k}}\right)$. As an example, for client *a* containing both image and text data, its modality combination is $X_{a}=\left(image,text\right)$, and its *i*-th local data sample is $X_{a}\left(i\right)={\left(x_{a}^{image},x_{a}^{text}\right)}_{i}$. Therefore, its modality number $M_{a}$ is 2.

In a communication round *t*, the local model $\theta_{k}^{t}$ of client *k* can be updated by a local training process via stochastic gradient descent (SGD):

(2) $$\theta_{k}^{t+1}=\theta_{k}^{t}-\mu \nabla L_{k}\left(X_{k},\theta_{k}^{t},y_{k}\right),$$

where μ is the learning rate of the local training process; $X_{k}$ is the corresponding local multimodal data; $L_{k}$ represents the total loss function of client *k* with multimodal input data $X_{k}$; and $\theta_{k}^{t}$ is the local model of client *k* parameters at communication round *t*.

Multiple modalities can make different contributions to the final loss affected by the problem context, data quality, and downstream tasks. For instance, in an image–text pair classification task, we may set a higher weight for the loss computed from image data and a lower one for text data. Therefore, given the input $X_{k}\left(i\right)$, the total loss $L_{k}$ is defined as

(3) $$L_{k}\left(X_{k}\left(i\right),\theta_{k}^{t},y_{k}\left(i\right)\right)=\sum_{j=1}^{M_{k}}\varphi_{k}^{m_{j}}l_{k}^{m_{j}}\left(C_{k}\left(x_{k}^{m_{j}};\theta_{k}^{t}\right),y_{k}\left(i\right)\right)$$

Here, $\varphi_{k}^{m_{j}}$ represents the sum weight of modality $m_{j}$; $C_{k}$ is the local model of client *k*; $l_{k}^{m_{j}}$ is the loss function for modality $m_{j}$; and $x_{k}^{m_{j}}$ is the input data of modality $m_{j}$.

Accordingly, we define the local training target as follows:

(4) $$f_{k}=\frac{1}{|D_{k}|}\sum_{i=1}^{|D_{k}|}L_{k}\left(X_{k}\left(i\right),\theta_{k},y_{k}\left(i\right)\right)$$

Thus, the global optimization target is defined as

(5) $$\min_{\theta_{G}}F\left(\theta_{G}\right)=\sum_{k=1}^{K}\omega_{k}f_{k}\left(\theta_{k}\right),$$

where $\theta_{G}$ is the global model parameters; $\omega_{k}$ is the global aggregation weight for client *k*; and *K* is the total number of clients.

## 5. Taxonomy of Multimodal Federated Learning

In [33], federated learning is categorized into horizontal federated learning, vertical federated learning, and federated transfer learning based on the data distribution characteristics. This previously proposed three-way division can clearly identify different categories of federated learning settings in unimodal scenarios.

However, it is not appropriate to directly adapt this categorization to multimodal federated learning. As the modality number of the local data expands, the distribution characteristics of the clients become more divergent. It is not illustrative enough to describe the data distribution relationship in such a way, especially when the clients contain different combinations of data modalities, i.e., the modality incongruity challenge proposed by [18].

For instance, in a mental health prediction task, three mobile users participating in federated systems may hold different preferences for digital APPs. As a result, their local datasets differ from data modalities and samples. There could exist the same data modalities, such as screen time, typing history, and common sensor data. The users can also hold unique modalities, such as image, video, audio, and APP data. In such a case, it is inappropriate to describe this federated system with horizontal federated learning or federated transfer learning alone.

In the abovementioned case, the relationships among clients could be decomposed into modality-wise levels. To describe the combined relationships as such, we extend the taxonomy by introducing *hybrid multimodal federated learning*. Thus, multimodal federated learning could be divided into four settings, which can be summarized as two categories based on the modality congruence, i.e., congruent MFL and incongruent MFL, as shown in Figure 2. Congruent MFL mainly covers horizontal settings, where all the clients hold the same modality set. Incongruent MFL, including vertical, transfer, and hybrid MFL, allows clients to have totally different or partially overlapped modality sets. We introduce the four categories in the below subsections and summarize them in Table 1.

### 5.1. Horizontal Multimodal Federated Learning

Similar to the unimodal horizontal setting, horizontal multimodal federated learning is defined as a multimodal distributed system where all the clients share the same modality combinations and the same data feature space but differ in terms of sample IDs.

**Definition 1** (Horizontal Multimodal Federated Learning). *Given a client set N and modality set M in a federated system, the system is called horizontal multimodal federated learning if, for ∀a,b∈N, the clients hold the same modality set, i.e., $|M_{a}\left|=\right|M_{b}|$ and $X_{a}=X_{b}$. Here, $|M_{k}|$ denotes the total number of modality types for client k, and $X_{k}$ is the modality combination set.*

**Figure 4:**
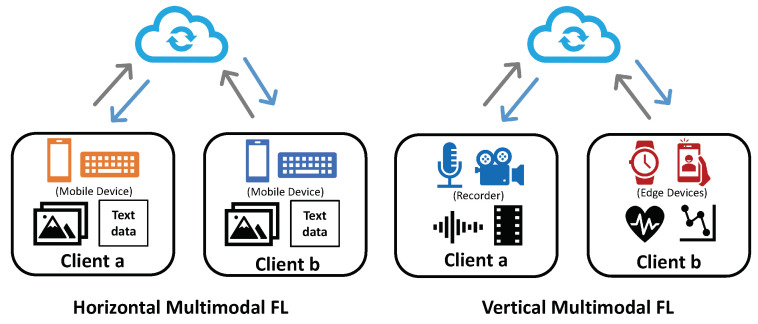
Illustration of horizontal multimodal federated learning and vertical multimodal federated learning. (**Left**): horizontal multimodal federated learning involving two clients. Both hold image and text data. (**Right**): the vertical multimodal federated learning example includes two clients with exclusive modalities. Client *a* has audio and video data, while client *b* holds heat rate and acceleration sensor data.

For instance, in Figure 4 (left), two mobile users, $X_{a}$ and $X_{b}$, with the same APP usage patterns can hold both image and text data (denoted as $x^{image}$ and $x^{text}$ modalities) on their devices, as shown in Figure 4 (left). With the same data modalities locally, the two clients have inputs that are the same in terms of the modality combination but different in terms of the sample IDs, mathematically defined as follows:

(6) $$X_{a}={\left\{{\left(x_{a}^{image},x_{a}^{text},y_{a}\right)}_{i}\right\}}_{i=1}^{|D_{a}|},X_{b}={\left\{{\left(x_{b}^{image},x_{b}^{text},y_{b}\right)}_{j}\right\}}_{j=1}^{|D_{b}|},$$

where ${\left(x_{a}^{image},x_{a}^{text},y\right)}_{i}$ denotes the *i*-th data sample of user *a* with two modalities, image and text, and the corresponding data label *y*, and $|D_{a}|$ represents the number of data samples.

In order to tackle the horizontal multimodal federated challenge in IoT systems, Zhao et al. in [34] proposed a generation-based method, which utilized an autoencoder as the feature extractor to support the downstream classifier on the server side. The authors also validated the effectiveness of their method in the missing modality challenge, where some clients only have certain shared data modalities in the horizontal federation. In [9], the authors used an ensemble of local and global models to reduce both data variance and device variance in the federated system.

### 5.2. Vertical Multimodal Federated Learning

Vertical multimodal federated learning defines a system with multiple unique data modality combinations held by different clients, where clients differ in terms of the feature space but share the same sample ID set. These modality combinations are also exclusive without an overlap in data modalities. These modalities could be aligned well in either spatial or temporal relationships.

**Definition 2** (Vertical Multimodal Federated Learning). *Given a client set N and modality set M in a federated system, the system is defined as vertical multimodal federated learning if, for ∀a,b∈N, they hold totally different modality combinations while connected by sample IDs, i.e., $X_{a}\cap X_{b}=\emptyset$ and $D_{a}=D_{b}$.*

For instance, in the human activity recognition task, a user may own multiple devices that collect different data modalities due to the divergence of the sensor category, as shown in Figure 4 (right). In a two-device case, the local datasets of the devices could be defined as:

(7) $$X_{a}={\left\{{\left(x_{a}^{video},x_{a}^{audio},y_{a}\right)}_{i}\right\}}_{i=1}^{\left|D\right|},X_{b}={\left\{{\left(x_{b}^{heart\_rate},x_{b}^{acceleration},y_{b}\right)}_{i}\right\}}_{i=1}^{\left|D\right|},$$

where client *a* holds modality video and modality audio, and modality heartratesensor and accelerationsensor are held by client *b*. Unlike the horizontal scenario, the two clients could share the same sample ID set D.

In [16], the authors proposed the feature-disentangled activity recognition network (FDARN) for the cross-modal federated human activity recognition task. With five adversarial training modules, the proposed method captured both the modality-agnostic features and modality-specific discriminative characteristics of each client to achieve better performance than existing personalized federated learning methods. Notably, each client held a single modality dataset that could differ from group to group in their experiments.

### 5.3. Multimodal Federated Transfer Learning

Multimodal federated transfer learning supposes that the clients in the federation have different input modalities and sample ID sets. Different clients are allowed to have some overlap in their modality combinations while differing in terms of the feature space. In other words, the clients may conduct different local tasks, such as VQA and image captioning for vision–language clients.

**Definition 3** (Multimodal Federated Transfer Learning). *Given a client set N and modality set M in a federated system, the system is defined as multimodal federated transfer learning if, for ∀a,b∈N, the clients hold different modality combinations and sample IDs, $X_{a}\cap X_{b}=\emptyset$ and $D_{a}\neq D_{b}$.*

**Figure 5:**
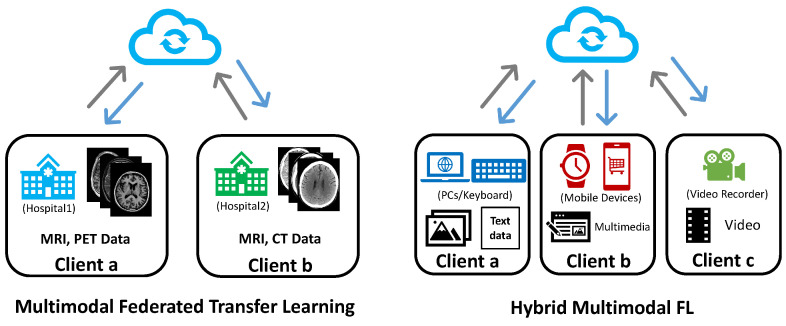
Illustration of multimodal federated transfer learning and hybrid multimodal federated learning. (**Left**): multimodal federated transfer learning involving two hospitals as clients. One holds MRI and PET data, the other holds MRI and CT data. (**Right**): hybrid multimodal federated learning including three clients with different modality combinations. The system contains both unimodal and multimodal clients.

As shown in Figure 5 (left), considering a federated learning example between different hospitals, a hospital located in a developed area usually takes equipment in advance compared to one in a rural area. In addition, due to the locations, the two hospitals may receive different patient groups. This may result in differences in their data modalities and the sample ID sets stored in their databases. For a two-party multimodal federated transfer learning system, the input modalities of the clients can be represented as

(8) $$X_{a}={\left\{{\left(x_{a}^{MRI},x_{a}^{PET},y_{a}\right)}_{i}\right\}}_{i=1}^{|D_{a}|},X_{b}={\left\{{\left(x_{b}^{MRI},x_{b}^{CT},y_{b}\right)}_{j}\right\}}_{j=1}^{|D_{b}|},$$

where the two clients differ in terms of both local data modalities and sample ID sets. However, since CT, MRI, and PET scans are all medical image techniques for diagnosis. The rich knowledge and model advantages could be shared between the clients, forming a typical multimodal federated transfer learning setting.

Liu et al. proposed aimNet to generate fine-grained image representations and improve the performance for various vision–language grounding problems under federated settings. They validated their methods in horizontal, vertical, and transfer multimodal federated learning settings to show their superiority.

### 5.4. Hybrid Multimodal Federated Learning

Hybrid multimodal federated learning is defined as a federated system where all the clients have incongruent data modalities in their local sets. The modality combination of each client is unique in the system, varying in terms of modality quantity and category. Both unimodal and multimodal clients can exist in the system.

**Definition 4** (Hybrid Multimodal Federated Learning). *Given a client set N and modality set M in a federated system, the system is defined as hybrid multimodal federated learning if there exist at least two basic relationships (horizontal, vertical, or transfer) or both unimodal and multimodal clients. There are at most $2^{M}-1$ types of clients in a hybrid federated system.*

In Figure 5 (right), we may take the mental health prediction task at the beginning of the section as an example of hybrid MFL, where three mobile users share a horizontally related screen time (ST) and hold a unique data modality. The input modalities of this example are

(9) $$X_{a}={\left\{{\left(x_{a}^{ST},x_{a}^{image},y_{a}\right)}_{i}\right\}}_{i=1}^{|D_{a}|},X_{b}={\left\{{\left(x_{b}^{ST},x_{b}^{video},y_{b}\right)}_{j}\right\}}_{j=1}^{|D_{b}|},X_{c}={\left\{{\left(x_{c}^{ST},x_{c}^{audio},y_{c}\right)}_{k}\right\}}_{k=1}^{|D_{c}|}.$$

The client category can vary in a hybrid setting. In a bimodal federated system, there could be three kinds of clients in total; for a trimodal federated system, the number of client categories could rise to seven depending on the different modality numbers and combinations. The relationships among the clients in a hybrid MFL system could be described at the modality level.

Chen and Zhang in [18] proposed FedMSplit, a dynamic and multiview graph structure aiming to solve the modality incongruity challenges in a hybrid MFL setting. The novel modality incongruity problem in MFL is a significant challenge within the scope of hybrid MFL. In [39], the authors proposed a general multimodal model that worked on both multitask and transfer learning for high-modality (a large set of diverse modalities) and partially observable (each task only defined on a small subset of modalities) scenarios. This indicates a recent research trend of designing more general-purpose multimodal models and reveals the importance of exploring hybrid MFL, the most challenging and complex multimodal federated scenario.

## 6. Tasks for Multimodal Federated Learning

Multimodal federated learning (MFL) offers many advantages, such as privacy preservation and addressing the data silo problem. However, it also faces limitations such as communication costs, data heterogeneity, and hardware disparities compared to centralized multimodal learning. Therefore, in addition to the unique challenges of modal heterogeneity, the original multimodal learning tasks become more challenging when performed within a federated learning framework. In this section, we will discuss several representative MFL application tasks.

### 6.1. Vision–Language Interaction

Visual and language data are widely present in data centers and edge devices, making vision–language interaction an important task in MFL. Specifically, a federated learning system targeting visual and language data should be capable of handling diverse and complex vision–language learning tasks, including visual question answering, visual reasoning, image captioning, image-text retrieval, and text-to-image generation. In the context of local training on client devices, the system needs to achieve efficient and robust multimodal matching and cross-modal interactions. On the one hand, due to constraints imposed by client hardware and communication requirements, lightweight and high-performance characteristics are desired in MFL approaches. Integrating state-of-the-art pre-trained large-scale models from the forefront of federated learning and vision–language learning has become a promising research direction. On the other hand, the heterogeneity of data, particularly in terms of labels and modalities, often leads to differences in model architectures and tasks among clients. These task gaps and semantic gaps can negatively impact global aggregation on the server side, posing challenges for achieving convergent global optimization.

Several pioneering studies have explored the field of MFL in the context of vision–language tasks. In [17], the authors proposed aimNet and evaluated it under horizontal FL, vertical FL, and federated transfer learning (FTL) settings when the clients were conducting either the VQA task or the image captioning task. CreamFL [37] utilized contrastive learning for ensembling uploaded heterogeneous local models based on their output representations. CreamFL [37] allowed both unimodal and multimodal vision–language tasks in federated systems. pFedPrompt [35] adapted the prompt training method to leverage large foundation models into federated learning systems to connect vision and language data. FedCMR [11] explored the federated cross-modal retrieval task and mitigated the representation space gap via weighted aggregation based on the local data amount and category number.

### 6.2. Human Activity Recognition

Wireless distributed sensor systems, such as IoT systems, where multiple sensors provide consistent observations of the same object or event, are a signification application scenario for MFL. Human activity recognition (HAR) is one of the most representative tasks in this setting, due to the privacy preservation requirement.

The data partition method for the HAR task in existing MFL works has two types, client-as-device and client-as-sensor. The former is represented by MMFed [10], which equally divides the multimodal data for each client. The local co-attention mechanism then performs multimodal fusion. Zhao et al. conducted their experiment by giving each client only a single type of modality [34]. The local network was divided into five modules for either modality-wise aggregation for clients with the same modality or general aggregation for all clients. However, the modality distribution or data partition method could vary according to hardware deployment and environmental factors.

### 6.3. Emotion Recognition

Emotion recognition plays a crucial role in improving social well-being and enhancing societal vitality. The multimodal data generated during the use of mobile phones often provide valuable insights into identifying users who may have underlying mental health issues. Effective emotion recognition algorithms can target specific users to enhance their experience and prevent the occurrence of negative events such as suicide and depression. However, multimedia data associated with user emotions, including chat records and personal photos, are highly privacy-sensitive. In this context, the MFL framework offers the capability of efficient collaborative training while ensuring privacy protection. Therefore, emotion recognition undoubtedly holds a significant position within the realm of MFL.

There are several MFL works that have investigated the emotion recognition task in the vertical and hybrid MFL setting. In [36], each client in the system held only one modality, and the unimodal encoders were trained on the local side. The proposed hierarchical aggregation method aggregated the encoders based on the modality type held by the clients and utilized an attention-based method to align the decoder weights regardless of the data modality. The FedMSplit approach [18] utilized a dynamic and multiview graph structure to flexibly capture the correlations among client models in a multimodal setting. Liang et al. in [40] proposed a decentralized privacy-preserving representation learning method that used multimodal behavior markers to predict users’ daily moods and identify an early risk of suicide.

### 6.4. Healthcare

Numerous healthcare centers and hospitals have accumulated vast amounts of multimodal data during patient consultations and treatments, including X-ray images, CT scans, physician diagnoses, and physiological measurements of patients. These multimodal data are typically tightly linked to patient identifiers and require stringent privacy protection measures. As a result, these healthcare institutions have formed isolated data islands, impeding direct collaboration in terms of co-operative training and data sharing through open databases. This presents a series of crucial challenges within the realm of multimodal federated learning, encompassing tasks such as AI-assisted diagnosis, medical image analysis, and laboratory report generation.

Some works in the field of healthcare have explored multimodal federated learning, often assuming that all institutions have the same set of modalities, referred to as horizontal MFL, or that each institution possesses only a single modality, known as vertical MFL. Agbley et al. in [14] applied federated learning for the prediction of melanoma and obtained a performance level that was on-par with the centralized training results. FedNorm [15] performed modality-based normalization techniques to enhance liver segmentation and was trained with unimodal clients holding CT and MRI data, respectively. Qayyum et al. utilized cluster federated learning for the automatic diagnosis of COVID-19 [13]. Each cluster contained healthcare entities that held the same modality, such as X-ray and ultrasound data.

## 7. Benchmarks for Multimodal Federated Learning

As discussed in Section 6, multimodal federated learning exhibits numerous broad application scenarios and tasks. However, the benchmarking of MFL frameworks specifically designed for testing and executing MFL tasks is still in its exploratory stage. Therefore, in this section, we present a series of benchmark datasets suitable for multimodal federated learning to facilitate further research endeavors.

### 7.1. Vision–Language Datasets

Caltech-UCSD Birds-200-2011 (CUB-200-2011). CUB-200-2011 [41] is one of the most widely used fine-grained categorization datasets. It contains 11,788 images of 200 subcategories belonging to birds. Each image has its own annotations for identification, which include one subcategory label, one bounding box, 15 part locations, and 312 binary attributes. Reed et al. expanded the dataset by providing ten fine-grained text description sentences for each image [42]. The sentences were collected through the Amazon Mechanical Turk (AMT) platform and had a minimum length of 10 words, without exposing the label and action information.

Oxford 102 Flower (102 Category Flower Dataset). Oxford 102 Flower [43] is a fine-grained classification dataset comprising 102 categories of flowers that commonly occur in the United Kingdom. Each category contains 40 to 258 images. There are 10 text descriptions for each image.

UPMC Food-101. Food-101 [44] is a noisy multimodal classification dataset that contains both images and paired captions of 101 food categories. Each category has 750 training and 250 testing images. There are a total of 101,000 images, each paired with one caption. However, the labels and captions of the training set contain some noise and may leak the label information. The testing set has been manually cleaned.

Microsoft Common Objects in Context (MS COCO). The MS COCO dataset [45] is a comprehensive dataset used for various tasks such as object detection, segmentation, key-point detection, captioning, stuff image segmentation, panoptic segmentation, and dense pose estimation. It comprises a total of 328 K images. The dataset provides detailed annotations for object detection (bounding boxes and segmentation masks), captioning, keypoint detection, stuff image segmentation, panoptic segmentation, and dense pose estimation. Note that the dense pose annotations are only available for training and validation images, totaling more than 39,000 images and 56,000 person instances.

Flickr30k. The Flickr30k dataset [46] comprises 31,000 images sourced from Flickr, accompanied by five reference sentences per image generated by human annotators. Additionally, we constructed an image caption corpus consisting of 158,915 crowd-sourced captions describing 31,783 images. This updated collection of images and captions primarily focuses on individuals participating in routine activities and events.

### 7.2. Human Activity Recognition Datasets

NTURGB+D120. The NTU RGB+D 120 dataset [47] is a comprehensive collection specifically designed for RGB+D human action recognition. It comprises a vast amount of data sourced from 106 unique subjects, encompassing over 114 thousand video samples and 8 million frames. This dataset encompasses a wide range of 120 distinct action classes, encompassing various activities that are part of daily routines, mutual interactions, and health-related actions. It serves as a valuable resource for research and development in the field of human action recognition, facilitating advancements in computer vision, machine learning, and artificial intelligence applications.

Epic-Kitchens-100. EPIC-KITCHENS-100 [48] is a large-scale dataset focusing on first-person (egocentric) vision. It features multi-faceted audio-visual recordings of individuals’ daily kitchen activities captured in their homes using head-mounted cameras. The dataset, comprising 45 kitchens across four cities, offers diverse environmental contexts. With 100 h of full-HD footage and 20 million frames, it provides a rich visual experience for analysis. The annotations, obtained through a unique ’Pause-and-Talk’ narration interface, enhance content understanding. The dataset includes 90,000 action segments and 20,000 unique narrations and supports multiple languages, facilitating cross-cultural studies. It covers a wide range of activities classified into 97 verb classes and 300 noun classes, enabling fine-grained analysis within the kitchen context.

Stanford-ECM. Stanford-ECM [49] is an egocentric multimodal dataset containing approximately 27 h of egocentric video recordings accompanied by heart rate and acceleration data. The video lengths vary from 3 min to around 51 min, ensuring a diverse range of content. The videos were captured using a mobile phone at 720×1280 resolution and 30 fps, while the triaxial acceleration was recorded at 30 Hz. A wrist-worn heart rate sensor captured heart rate readings every 5 s, and the phone and heart rate monitor were synchronized via Bluetooth. All data were stored in the phone’s storage, with any gaps in heart rate data filled using piece-wise cubic polynomial interpolation. The data were meticulously aligned to the millisecond level at a frequency of 30 Hz, ensuring precise synchronization across the modalities.

mHealth (Mobile Health). The mHealth (Mobile Health) dataset [50] is a collection of body motion and vital sign recordings from ten volunteers engaging in various physical activities. The dataset includes sensors placed on the chest, right wrist, and left ankle to measure acceleration, rate of turn, and magnetic field orientation across different body parts. Additionally, the chest sensor provides two-lead ECG measurements, allowing for potential applications in basic heart monitoring, arrhythmia detection, and the analysis of exercise effects on the ECG. Overall, the dataset consists of 12 activities performed by 10 subjects, with three sensor devices utilized for data collection.

### 7.3. Emotion Recognition Datasets

Interactive Emotional Dyadic Motion Capture (IEMOCAP). The IEMOCAP database [51] is a multimodal and multispeaker dataset designed for studying emotional expressions. It encompasses around 12 h of audiovisual data, including video recordings, speech, facial motion capture, and text transcriptions. The database features dyadic sessions in which actors engage in improvised or scripted scenarios carefully crafted to elicit emotional responses. Multiple annotators have labeled the IEMOCAP database with categorical labels like anger, happiness, sadness, and neutrality, as well as dimensional labels such as valence, activation, and dominance.

Multimodal Corpus of Sentiment Intensity (CMU-MOSI). The CMU-MOSI dataset [52,53] consists of 2199 opinion video clips, each annotated with sentiment values ranging from −3 to 3. The dataset includes detailed annotations for subjectivity, sentiment intensity, visual features annotated per frame and per opinion, and audio features annotated per millisecond.

CMU Multimodal Opinion Sentiment and Emotion Intensity (CMU-MOSEI). The CMU-MOSEI dataset [52,53] is a multimodal data collection for analyzing sentiment and emotion in opinionated text and speech. It contains a total of 23,453 video segments extracted from 1000 YouTube videos, with each segment accompanied by transcriptions and audio and visual features. The dataset provides rich annotations for sentiment, emotion, and intensity, allowing researchers to explore the interplay between language, speech, and visual cues in expressing opinions and emotions.

### 7.4. Healthcare Datasets

MIMIC-IV. The Medical Information Mart for Intensive Care (MIMIC-IV) dataset [54] is a comprehensive and widely used database that provides detailed clinical data from patients admitted to intensive care units (ICUs). MIMIC-IV offers an expanded collection of de-identified electronic health records (EHRs) from diverse healthcare institutions. It contains a wealth of information, including vital signs, laboratory results, medications, procedures, diagnoses, and patient demographics. The dataset is invaluable for conducting research and developing algorithms and models related to critical care medicine, clinical decision making, and healthcare analytics.

MIMIC-CXR. The MIMIC Chest X-ray (MIMIC-CXR) database [55] is a large, publicly available collection of de-identified chest radiographs in DICOM format accompanied by corresponding free-text radiology reports. It comprises 377,110 images from 227,835 radiographic studies conducted at the Beth Israel Deaconess Medical Center in Boston, MA. The dataset adheres to the US Health Insurance Portability and Accountability Act of 1996 (HIPAA) Safe Harbor requirements, ensuring the removal of protected health information (PHI). Its purpose is to facilitate diverse medical research areas, including image analysis, natural language processing, and decision support, providing a valuable resource for advancing knowledge and innovation in the field of medicine.

Alzheimer’s Disease Neuroimaging Initiative (ADNI). The Alzheimer’s Disease Neuroimaging Initiative (ADNI) database [56] is a comprehensive and widely used collection of data aimed at advancing research in Alzheimer’s disease and related neurodegenerative disorders. ADNI consists of clinical, genetic, imaging, and biomarker data gathered from participants across multiple sites in the United States and Canada. The dataset includes various modalities such as magnetic resonance imaging (MRI), positron emission tomography (PET), cerebrospinal fluid (CSF) biomarkers, and cognitive assessments.

### 7.5. Multisensor Datasets

ModelNet40. The ModelNet40 dataset [57] comprises 3D synthetic object point clouds, making it a highly utilized benchmark in point cloud analysis due to its diverse categories, precise shapes, and well-organized dataset. In its original form, ModelNet40 encompasses 12,311 CAD-generated meshes representing 40 categories, including objects like airplanes, cars, plants, and lamps. Out of these, 9843 meshes are designated for training purposes, while the remaining 2468 meshes are set aside for testing. The corresponding point cloud data points are uniformly sampled from the surfaces of these meshes and subsequently preprocessed by repositioning them to the origin and scaling them to fit within a unit sphere.

Vehicle Sensor. The Vehicle Sensor dataset [58] was proposed for the vehicle type classification task in wireless distributed sensor networks (WDSN). The dataset consists of 23 road segmentation instances. Each instance has 50 acoustic and 50 seismic features.

### 7.6. Multitask Dataset

FedMultimodal. FedMultimodal [59] was the first federated learning benchmark designed for multimodal learning, encompassing five key multimodal applications across ten well-known datasets, featuring a total of eight distinct modalities. This benchmark introduces a comprehensive FL pipeline, enabling a holistic modeling framework that covers data partitioning, feature extraction, FL benchmark algorithms, and model evaluation. In contrast to existing FL benchmarks, FedMultimodal offers a standardized methodology for evaluating FL’s resilience in real-world multimodal scenarios, specifically addressing three prevalent data corruption types: missing modalities, missing labels, and erroneous labels.

## 8. Discussion

We introduce the potential directions and challenges of multimodal federated learning in this section. These challenges are non-exclusive; rather, they are rooted in one core factor, the data modality distribution in the federated learning system.

### 8.1. Modality Heterogeneity

The heterogeneity problem in unimodal FL is usually caused by the imbalance of data quantity and data label skews. While the introduction of modality distribution will further increase the complexity of the problem, the heterogeneity in both statistical distribution and modality distribution will affect the global convergence and performance of the federated system. In addition, most existing non-IID and personalized methods are questionable in their effectiveness in multimodal federated settings. Thus, innovative and effective solutions are expected to be proposed for this setting.

In the MFL setting, the modality heterogeneity challenge exists at both the client level and the system level. At the client level, the clients require efficient local representation learning to bridge the semantic gaps [60,61] among the multimodal data. One of the most popular solutions for multimodal representation learning is to map the different modalities of data onto a mutual latent space. Similar to centralized multimodal learning, how to adapt advanced knowledge from representation learning and centralized multimodal learning to design feature extractor modules for merging these gaps in the local learning process is a vital topic.

At the system level, there exists a task gap among all the clients caused by the differences in modality combination, e.g., the modality types in the local datasets. In centralized multimodal learning, representation learning usually transforms the different modalities into a common representation space via an embedding operation. Comparatively, MFL divides the common space of the centralized scenario into *N* common subspaces, making the unification of the embedding operation among all the clients difficult. A client maps the original multimodal data onto embedding representations, which all exist in its unique common subspace. Due to the modality heterogeneity and the different local model tasks, these common subspaces differ from each other, resulting in the task gaps that are difficult to bridge. For example, unimodal clients and multimodal clients could hold totally different parameter spaces and work on different feature spaces. Even if the clients hold the same modality combinations, the specific local tasks can be different, such as visual question answering and image captioning.

To solve this challenge, it is necessary for a new aggregation paradigm. The modality heterogeneity could result in more divergent gradients and even heterogeneous local model architectures. As Wang et al. proved that different modalities overfit and generalize at different rates, the one-fits-all global training strategy for MFL might not work, since optimizing all the clients jointly can produce suboptimal results [62].

### 8.2. Missing Modalities

Another significant challenge for MFL is missing modalities, referring to some clients suffering a data quantity imbalance among the different modalities of their local datasets. For instance, for a client that holds 1000 image–text data pairs, 300 of the pairs might lose their image data and 200 of them might not have the text part. The absence of modalities poses challenges for both the structure and robustness of a model. Many transformer-based models [63] will encounter significant performance degradation in such cases.

Missing modalities occur frequently in realistic scenarios due to hardware limitations, collection errors, and storage issues. To address the issue of missing modalities, some strategies and techniques for centralized learning have been proposed. Some approaches [64,65] involve data imputation or reconstruction methods to fill in the missing modalities based on the available information. Others leverage multitask learning or meta-learning [63] techniques to transfer knowledge from modalities with sufficient data to those with missing data. Additionally, there have been efforts to design more robust and flexible models [66] that can effectively handle missing modalities without significant performance degradation. In regard to these developments in addressing missing modalities in centralized learning, it is crucial to explore lightweight and data-efficient methods for MFL to tackle this challenge.

### 8.3. Data Complexity

Multimodal federated learning serves as a promising solution for collaborative ML-based systems among healthcare entities and medical-related institutions. On the one hand, medical research and patient diagnosis generate massive multimodal data, which is a great resource to boost the development of advanced ML methods. On the other hand, these healthcare data, such as electronic health records (EHRs) and X-ray and CT scan images, are stored and managed in a privacy-sensitive manner. MFL enables the exploration of information within such complex data, facilitating collaborative training across healthcare and medical data silos and thereby delivering improved AI-assisted diagnosis, medical image analysis, report generation, and other related services.

In contrast to applications in the IoT or multimedia domains, healthcare data are more complex and diverse in terms of both format and granularity. The heterogeneity of the data is further amplified within healthcare institutions due to differences in medical equipment, diagnostic methods, and data management practices, making federated collaboration [67] more difficult. A large amount of medical-related MFL work has emerged. Cobbinah et al. in [68] provided an FL-based method for the prediction of the phase of Alzheimer’s disease utilizing MRI data from multiple centers. In [13,14], the authors used MFL for AI-assisted disease diagnosis and achieved satisfying performance. FedNorm [15] explored medical image segmentation in the FL setting.

### 8.4. Large-Scale Pre-Trained Models

Among the recent progress made in multimodal learning, the inspiring performance of large-scale pre-trained models [69,70,71] indicates a promising future for solving broad machine learning tasks with a unified and effective solution. However, there still exist two main challenges known to block the universal deployment of these large-scale pre-trained models in federated learning systems.

On the one hand, the cost of building and training these large-scale pre-trained models can be extremely high and unaffordable for most computing devices and data centers. On the other hand, large amounts of training data must be gathered for the effective training of large foundation models. In a federated learning system, the clients usually have limited hardware resources and communication bandwidth, which makes it impractical to train from scratch or even fine-tune large-scale foundation models in FL scenarios. On the other hand, utilizing traditional knowledge distillation methods to transfer knowledge from pre-trained large models within the framework of federated learning also faces limitations. This is due to the fact that the training data for pre-training large models are often abundant and diverse, making it challenging for clients in a federated learning system to collect and store such data. These existing limitations hinder the deployment and empowerment of large-scale foundation models on distributed learning frameworks represented by federated learning. The ability to integrate large-scale pre-trained models in a lightweight way is expected for an effective federated multimodal learning framework.

To overcome these limitations, several works have been proposed to tackle the deployment of large-scale pre-trained models in multimodal federated learning. FedBERT [72] adapted split learning to achieve efficient distributed training for the large-scale BERT [69] model. The head and mapping layers of BERT were distributed to clients for local training and then aggregated on the server side. Ref. [73] proposed the federated prototype-wise contrastive learning (FedPCL) algorithm, which used class prototypes and contrastive learning to share class-relevant knowledge among clients, demonstrating improved personalization and knowledge integration capabilities with a pre-trained backbone model. FedCLIP [38] added an adapter module after the CLIP backbone to achieve the efficient deployment of the CLIP model [70] with federated clients. Some studies [19,35] have utilized the idea of prompt training to aggregate the user consensus via a learnable prompt and improve the users’ characteristics in the visual domain. Improving the ability to integrate large-scale pre-trained models will greatly enhance the performance of MFL systems.

### 8.5. Privacy Concerns

Multimodal data increase the possibility of user identification in federated learning systems due to the complementary information among various modalities. Multimodal fusion methods, represented by late fusion, increase the risk of inference attacks [74,75]. For example, malicious users could infer other modalities from a certain modality.

Compared to traditional unimodal federated learning, applying traditional defense methods, such as differential privacy [76] and encrypted transmission [77,78,79], to multimodal systems is more challenging. Different modalities may provide different levels of information granularity, making it more difficult for differential privacy to strike a satisfying trade-off between privacy protection and performance. Meanwhile, in incongruent MFL, modality heterogeneity may lead to significant differences in local model structures among different clients, posing a risk of information leakage during aggregation. Therefore, federated aggregation methods based on model distillation and heterogeneous model recombination could be promising solutions for privacy leakage risk in MFL. Considering the aforementioned concerns, it is important to pay more attention to privacy protection for MFL. Liang et al. in [40] conducted an early exploration of privacy protection techniques for multimodal federated learning. In their mood prediction task using mobile data, they adopted adversarial training and orthogonal decomposition methods to remove the sensitive information features when communication occurred.

### 8.6. Weakly Supervised Learning

In realistic application scenarios, the multimodal data collected in data silos usually contain limited supervision signals such as labels or matching relationships among the modalities. The exploration of weakly supervised learning in MFL is another crucial topic, which also includes self-supervised learning and semi-supervised learning.

Some unimodal FL studies have investigated the field of self-supervised learning [80,81] and semi-supervised learning [7,82,83,84] techniques in FL. These studies focused on scenarios in which the system contains no or limited data labels. However, the heterogeneity of multimodal data will further challenge the robustness of systems with limited and noisy supervision signals. On the one hand, in situations where there are significant differences in modality combinations among clients, it is difficult to share the knowledge of unsupervised learning through the aggregation of model parameters. On the other hand, multimodal data may have a matching issue, where data belonging to the same image–text pair exist in local datasets with different data IDs. This could lead to confusion in the alignment relationships between representations, as exemplified by contrastive learning methods.

These directions hold great potential for advancing the field of multimodal federated learning by enabling models to learn from diverse and abundant but weakly labeled data sources, thus paving the way for improved performance and generalization in real-world applications.

## 9. Conclusions

In this paper, we delved into the promising research area of multimodal federated learning (MFL). We provided an introduction to existing MFL methods and discussed the motivations behind leveraging distributed multimodal data. Recognizing that many studies in this domain have proposed customized scenario settings, we took the initiative to formally define multimodal federated learning and categorize and organize existing works. Our aim was to establish standards for subsequent research and foster a coherent and structured approach in this evolving field. Addressing the lack of evaluation and benchmarking, we refined several representative MFL application scenarios and identified relevant datasets. These efforts will allow the research community to compare and analyze task performance, ultimately promoting advancements in MFL. Moreover, we emphasized the core issue of modality heterogeneity, which presents unique challenges to MFL, including dealing with missing modalities and the deployment of pre-trained models. Additionally, traditional privacy protection and data heterogeneity have become more complex in MFL. By highlighting these challenges, we sought to raise awareness among researchers and encourage innovative solutions. Overall, this survey paper provides preliminary summaries and explorations that can significantly contribute to a better understanding of the importance and uniqueness of the MFL field. We hope our insights will serve as valuable guidance for researchers and inspire further development in this promising area of research.

## Figures and Tables

**Table 1:** Taxonomy of multimodal federated learning based on input modalities.

Category	Characteristics	Example	Related Work
Horizontal	Same input modalities, same feature space, different sample ID space.	Mobile phone users who use similar apps.	[9,11,14,15,17,20,34,35]
Vertical	Different input modalities, different feature space, same sample ID space.	IoT devices from different companies owned by a single user.	[13,16,36]
Transfer	Different input modalities, different feature space, different sample ID space.	Federated collaboration between healthcare centers with different socioeconomic conditions and locations.	[17,37,38]
Hybrid	Mixed combinations of different, partially different, or even the same input modalities, feature space, and sample ID space.	Mental health prediction task with diverse mobile users.	[12,15,18,39]

## Data Availability

Not applicable.
